# Fumarase mediates transcriptional response to nutrient stress

**DOI:** 10.15698/cst2017.10.107

**Published:** 2017-10-01

**Authors:** Qin Zhao, Yuhui Jiang

**Affiliations:** 1 The Institute of Cell Metabolism, Shanghai Key Laboratory of Pancreatic Disease, Shanghai General Hospital, Shanghai Jiaotong University, School of Medicine, Shanghai 200080, China.

**Keywords:** fumarase, cellular response, nutrient condition

## Abstract

Limited supply of nutrient normally causes cell growth arrest. Our recent study (Nat Cell Biol. (7):833-843) shows that fumarase (FH), a key enzyme responsible for the conversion between fumarate and malate in tricarboxylic acid cycle, is importantly involved in the cellular response to nutrient condition.

 To date, emerging evidence demonstrate that metabolites participate in a wide variety of cellular activities besides their direct role in metabolism. Previous studies have reported that metabolic activity of FH is critically involved in the precise regulation of histone 3 lysine 36 dimethylation (H3K36me2) upon DNA damage, which is based on the inhibitory effect of fumarate on α-ketoglutarate-(α-KG)-dependent histone demethylase KDM2A/B. Strikingly, through mass spectrum and immunoprecipitation analysis, here we first found that glucose deprivation largely promoted FH binding to ATF2, a canonical transcription factor responsive to stress signaling. Further ChIP analysis show that ATF2 recruits FH to the promoter regions of its targeted genes for cell cycle arrest. Accordingly, gene expression and functional analysis demonstrate that the ATF2-FH complex facilitates transcription and thus cell cycle arrest under glucose deficiency.

Subsequently, we performed a sequence of biochemical analyses and found that in normal pancreatic duct cells glucose deprivation readily triggered FH Ser75 phosphorylation by AMPK. This phosphorylation is required for FH interaction with ATF2 and the subsequent gene expression. In contrast, FH-ATF2 complex formation is weakly detected in pancreatic duct cancer cells under glucose deprivation, implying that a negative regulation for AMPK-FH-ATF2 signaling exists. After screening, we found protein O-GlcNAcylation, which is tightly related to glucose availability and is reversely catalysed by O-GlcNAc transferase (OGT) and O-GlcNAcase (OGA), implicated in this process. Intriguingly, mass spectrum and *in vitro *O-GlcNAcylation assay indicate that FH is O-GlcNAcylated by OGT at Ser 75; and cancer cells exhibit high FH O-GlcNAcylation levels along with a strong OGT activity, which soundly impairs AMPK-mediated FH phosphorylation and the relevant cellular sensitivity to the low glucose availability.

To understand the metabolic effects of FH on transcription, FH enzymatic dead mutant FH R233H and ATF2 binding defect mutant FH S75A were both utilized and the results indicate FH catalytic activity as well as its binding to ATF2 are both indispensable for ATF2-mediated transcription. Meanwhile, the importance of FH metabolic activity is validated by addition of exogenous fumarate which partially reverses the repressive effect of FH R233H and FH S75A on transcription and cell cycle arrest. Furthermore, the implication of locally generated fumarate is expanded to its epigenetic effect on H3K36me2 regulation; indirect evidence from sequential biochemical analyses exemplify that fumarate produced by promoter-associated FH, maintains H3K36me2 levels via inhibition of α-KG-dependent histone demethylase KDM2A directly at the promoter region, in which H3K36me2 level is positively related with the transcription of genes targeted by ATF-FH complex (**Figure 1**).

**Figure 1:**
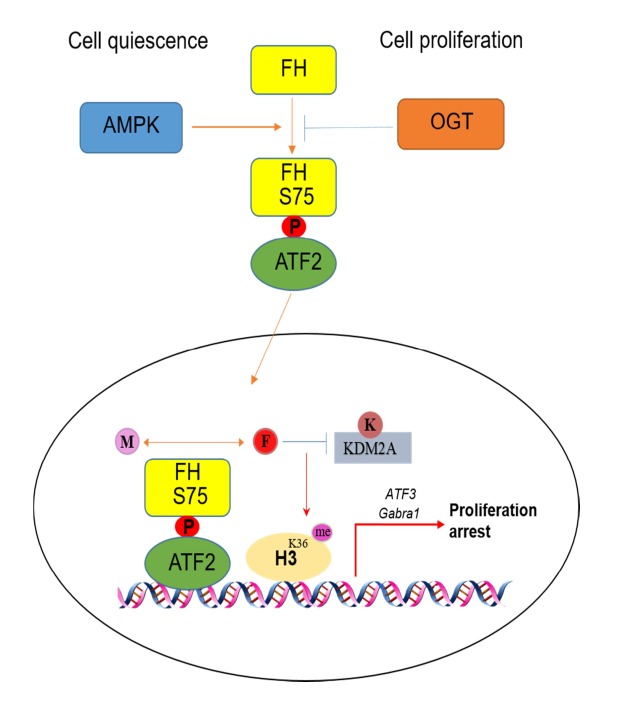
FIGURE 1: A schematic model of transcriptional regulation by FH in cell quiescence and proliferation status under glucose deprivation. M: malate; F: fumarate; K: α-KG.

At the physiological level, the FH S75A mutant significantly rescued the impaired xenograft tumor development resulted by OGT depletion, which indicates competition of OGT-mediated FH O-GlcNAcylation to FH Ser75 phosphorylation would be essential for cancer cell growth under condition of glucose deficiency *in vivo*. In consistence, clinical data show FH Ser75 phosphorylation is inversely correlated with OGT levels in human pancreatic tumour specimens, and patients with higher FH Ser75 phosphorylation display a longer median survival duration.

The interplay between metabolism and epigenetic regulation are fundamentally involved in cellular response to microenvironmental nutrient status. Our study focuses on the subtle regulation of histone methylation-gene transcription by FH under glucose deficiency-induced signaling pathway. The flexible functional role of FH reflected here is attributed to the counteractive effect of OGT activity on AMPK signaling; this finding would valuably provide new molecular basis for developing effective cancer therapy in future.

